# Tomographic comparison of the cochlea, oval window, round window and facial nerve between adults and children and their influence on cochlear implant surgery

**DOI:** 10.1016/j.bjorl.2025.101731

**Published:** 2025-10-24

**Authors:** Rogerio Hamerschmidt, Mohamad Feras Al-lahham, Bettina Carvalho, Mayara Risnei Watanabe, Rogério de Azevedo Hamerschmidt, Isadora Mansur Castro

**Affiliations:** a Universidade Federal do Paraná (UFPR), Departamento de Otorrinolaringologia, Curitiba, PR, Brazil; b Universidade Federal do Paraná (UFPR), Curitiba, PR, Brazil

**Keywords:** Cochlear implants, Cochlea, Tomography

## Abstract

**Objective:**

Is to compare two tomographic measurements that can be used to predict the visibility of the round window: the angle formed by the junction of the line that goes from the round window Membrane Center Point (MJR) to the Facial Nerve surface (FN) with the Coronal Plane (CP) traced over this surface (RWM-FN-CP Angle) and the vertical distance between the midpoint of the RWM and this Coronal Plane (RWM-CP Vertical Distance); in adults and children, in order to verify if there are significant differences between the groups, which would justify the greater surgical difficulty caused by the lower visibility of the RW in children.

**Methods:**

In this retrospective study, the RWM-FN-CP angle and the RWM-CP vertical distance were measured in Computerized Tomography Scans (CT) of patients who underwent CI surgeries.

**Results:**

32 individuals who underwent CT in the period 2015–2018, 16 adults aged 27–73 years, and 16 children, with a mean age of 3.4 (±2.7) years were evaluated. The mean of the RWM-FN-CP angle in children was significantly lower, than the mean in adults. The mean RWM-CP vertical distance in children was significantly lower than the mean in adults (p = 0.0001).

**Conclusion:**

In children, the RWM-FN-CP angle is more acute, and the measured MJR-PC vertical distance is smaller when compared to adults. The anatomical differences observed in the tomographies justify the lower visibility of the RW during the surgical act of cochlear implantation in children.

**Level of evidence:**

3.

## Highlights

• Cochlear implant surgery via round window ensures less trauma.
• Understanding morphology and anatomical relationships of the round window is important.
• Angle between Round Window Membrane (RWM) and the Facial Nerve (FN) with the Coronal Plane (CP).
• Vertical distance between Round Window Membrane (RWM) and the Facial Nerve (RWM-FN).
• RWM-FN-CP angle is more acute, and the vertical distance (RWM-FN) is smaller in children.

## Introduction

About 50 million of adults suffer from profound or severe deafness worldwide, where there is a level of deficiency not well corrected with hearing aids. Although the cause of hearing loss is variable, most share a common pathway: damage or absence of sensory hair cells in the cochlea. This leads to the inability to transmit the acoustic signal to the auditory nerve and, consequently, there is no processing of this signal by the central nervous system.^1^ Among the main causes of sensorineural hearing loss, the following stand out like damage or loss of hair cells due to aging, noise exposure, ototoxic drugs, infection or genetic abnormalities; degeneration of spiral ganglion cells due to primary or secondary causes; asynchronous firing, congenital damage, loss or absence of the cochlear nerve; temporal lobe dysfunction or damage or impaired central auditory processing.

Because they are not treatable with hearing aids, most of these cases could benefit from the Cochlear Implant (CI). CI surgery is the most effective method in the treatment of severe to profound deafness, both in adults and children. The classic surgical approach is made through a mastoidectomy followed by a posterior tympanotomy to make the opening of the Facial Nerve (FN) recess, and the visualization of the RW. The surgeon can choose between inserting the electrode through the RW or through a cochleostomy, where the promontory is perforated.^2^

It has been previously suggested that the perceived angle of the RW affects the trauma of electrode insertion, such that the more posteriorly oriented the RW, the greater the likelihood of atraumatic electrode insertion with inherent implications for hearing preservation.^3^

Historically, it was believed that the morphology and spatial orientation of the labyrinth did not change significantly after birth. Now, scientific evidence has shown that skull growth during the early years and puberty can impact the mastoid process and the tympanic and squamous portions of the temporal bone, as well as change the orientation of the cochlear basal turn in relation to the Facial Recess (RF).^4^^,^^5^ Thus, there may be differences in the visibility of the (RW) according to the patient's age group.

As for complications, studies report more complications in adults than in children.^6^^,^^7^ Hansen, farinetti we considered if these possible complications would be related to the positioning of the RW, which would in turn make the insertion of the CI electrode more difficult.

Therefore, the present work proposes to compare the anatomical measurements of RW (the angle of the Round Window Membrane, the Facial Nerve in the Coronal Plane [RWM-FN-CP] and the vertical distance between the Round Window Membrane and the Facial Nerve [RWM-FN]), in adults and children, based on Computed Tomography (CT).

## Methods

IRB aproval was obtained.

### Tomographic measurements

High resolution ear CT scans of patients were evaluated.

On these scans, angles and pertinent anatomy were manually delineated and measured blinded as to the age of the patient.

To assess the angle between the facial nerve and the round window, a protocol based on high-resolution tomographic images was used, with slice thicknesses between 0.5 mm and 0.6 mm. Initially, a coronal plane was defined at the level of the facial nerve, using the orbitomeatal line and the Temporomandibular Joint (TMJ) as anatomical references. From this coronal plane, the following steps were performed: In the axial plane, the perpendicular distance between the previously defined coronal plane and the round window was measured. The angle formed between the facial nerve and the round window was traced and measured in this same axial plane.

Only tools of the Picture Archiving and Communication System were used.

### Surgical approach

For implant placement, there are two possible approaches: via cochleostomy or via RW. The methodology via cochleostomy involves perforating the promontory to fix the implant, which is not necessary via RW. Thus, the RW method has less trauma, in addition to less bone and perilymph loss, when compared to cochleostomy. In view of this, the RW method results in less neural tissue degeneration, ensuring preservation of inner ear structures.^2^^,^^7^

The surgical approach can be done under general anesthesia, or local anesthesia with sedation.^9^

### Study variables

**Fig. 1:**
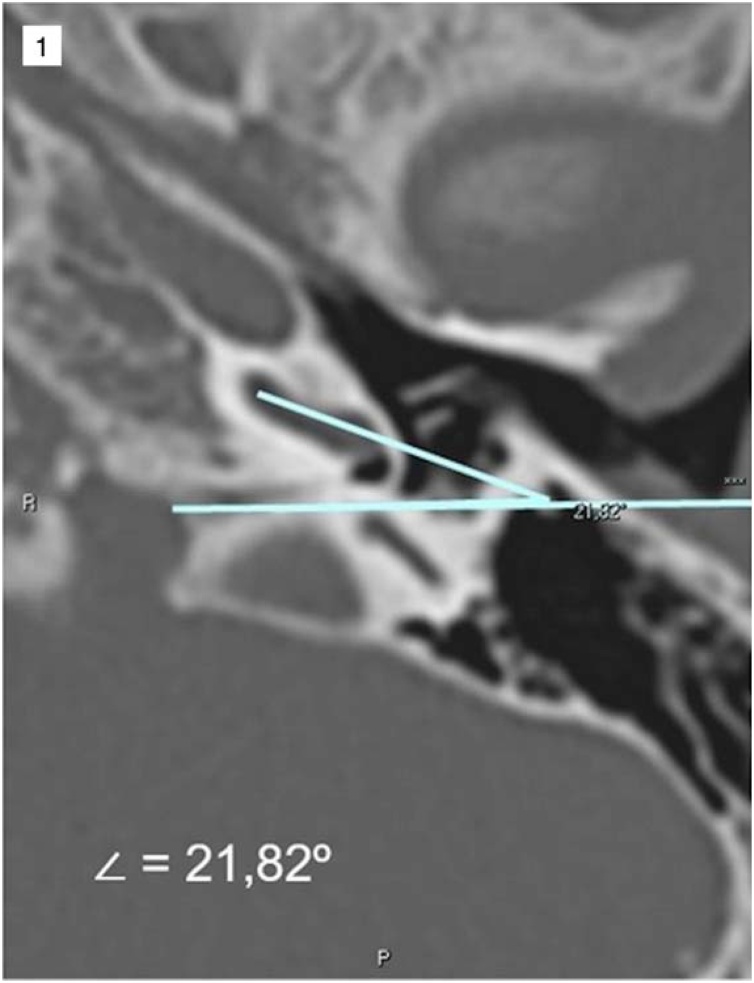
Example of round window membrane angle measurement, facial nerve in the coronal plane (RWM-FN-CP). The picture shows a line that passes tangent to the round window niche (anterior lip), since round window size and shapes may vary the radiologist used this bony landmark as other tomography-based works.

**Fig. 2:**
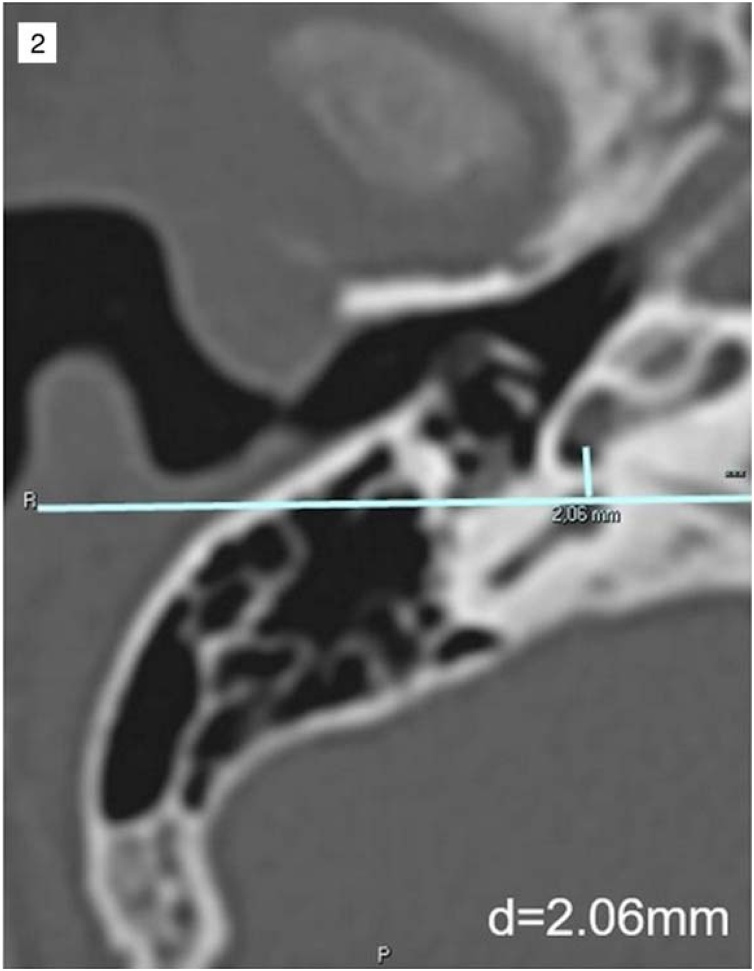
Example of vertical distance measurement between the Round Window Membrane and the Facial Nerve (RWM-FN). The line passing through the ampulla of the posterior semicircular canal is at the same height as the facial nerve.

At the time of the procedure, measurements of the angle of the Round Window Membrane, the Facial Nerve in the Coronal Plane (RWM-FN-CP) (Fig. 1) and the vertical distance between the Round Window Membrane and the Facial Nerve (RWM-FN) were evaluated (Fig. 2) bilaterally and in the axial tomographic section with greater visibility of the round window membrane.

All measurements were done by the same radiologist, using software program.

### Statistical analysis

The normality was tested for the RWM-FN-CP angle and RWM-CP vertical distance variables using the Kolmogorov-Smirnov normality test and Levene's homogeneity of variance test. Taking into account the results presented for the choice of statistical tests, comparisons between groups in relation to the RWM-FN-CP angle and RWM-CP vertical distance by right measurement were performed using the Mann-Whitney test, while the data per left measurement were analysed using unpaired Student's *t*-test. The significance level adopted was α < 0.05. Graphs were created using the GraphPad Prism v.8.4.0 software.

## Results

In this study, 32 patients who underwent CT in the period of 2015‒2018 at the Otorhinolaryngology Service of the Hospital de Clínicas, Universidade Federal do Paraná (Table 1) were included, being 16 adults aged 27–73-year-old, and 16 children, with a mean age of 3.4 (±2.7) years old (Table 2).

**Table 1:** Data related to patients included in the study with right and left measurements of vertical distance RWM-CP and angle RWM-FN-CP.

		Right measurement		Left measurement				Left measurement		Left measurement	
Adults	Age	Distance (mm)	Angle (°)	Distance (mm)	Angle (°)	Children	Age	Distance (mm)	Angle (°)	Distance (mm)	Angle (°)
1	44 + 5 m	3.9	34	4.3	37	1	2 + 11 m	2.0	24	2,0	21
2	44 + 9 m	2.7	24	4.1	36	2	3 + 3 m	3.6	30	3,1	31
3	61 + 5 m	3.4	28	3.3	32	3	2 + 8 m	1.9	18	3,1	28
4	37 + 3 m	3.7	28	4.5	34	4	2	3.9	32	3,3	24
5	60	4.0	33	4.2	33	5	1 + 8 m	2.5	25	3,5	32
6	41 + 10 m	4.1	34	4.5	41	6	2 + 7 m	2.1	19	3,8	36
7	38	3.1	27	3.3	30	7	0 + 11 m	1.8	16	2,6	23
8	27	5.1	36	3.8	29	8	0 + 8 m	3.8	32	5,5	41
9	47	3.5	31	4.2	37	9	10	3.8	35	4,0	32
10	51	3.6	30	3.8	29	10	9	3.2	23	2,7	26
11	28	3.7	33	3.3	32	11	2 + 6 m	1.9	18	3,1	28
12	41	3.3	29	4.8	39	12	2 + 9 m	2.0	24	2,0	21
13	73	4.4	36	4.5	38	13	1	1.8	16	2,6	23
14	42 + 4 m	4.1	34	4.6	41	14	3 + 1 m	3.6	30	3,1	31
15	59	4.0	33	4.2	33	15	6 + 2 m	3.6	35	3,8	32
16	46 + 7 m	3.9	34	4.3	37	16	2 + 10 m	2.2	19	3,6	36

Caption: Age represented in years + months (m); vertical distance RWM-CP represented in millimeters (mm); angle RWM-NF-CP represented in degrees (º).

**Table 2:** Descriptive analysis and age of the different groups evaluated.

Group	n	Average	SD	Min	Median	Max
Adults	16	46.5	12.3	27	44.7	73
Children	16	3.4	2.7	8^a^	8	10

Notice: Age values are represented in years, with the exception of the minimum age data for children (^a^), represented in months. n, sample Number; SD, Standard Deviation; Min, Minimum age found in the group; Max, Maximum age found in the indicated group.

### RWM-FN-CP angle

**Fig. 3:**
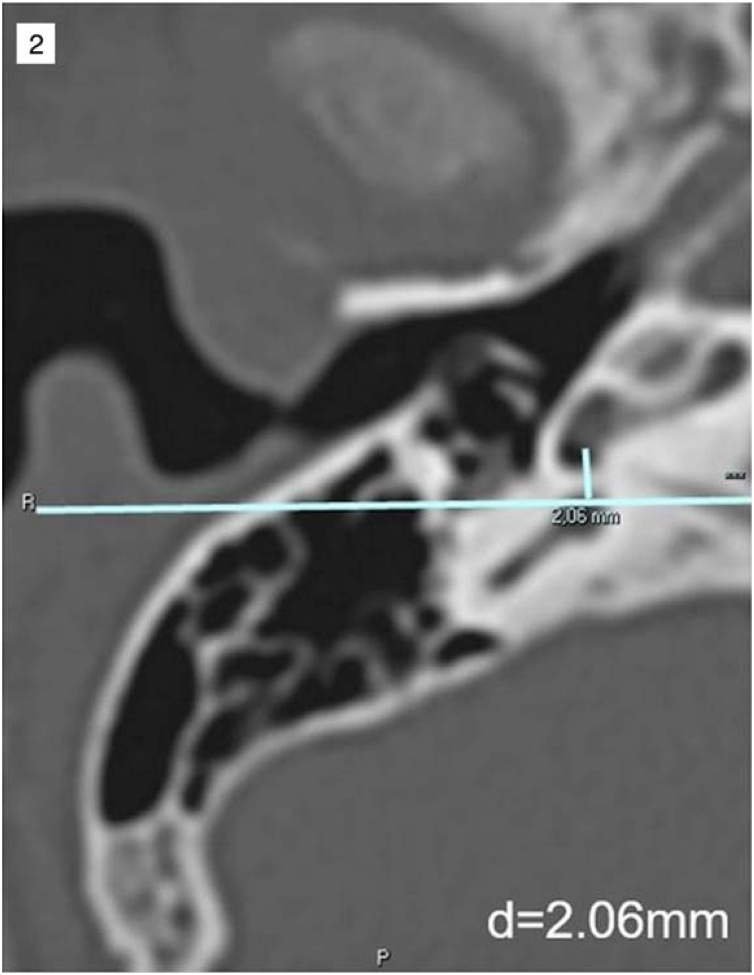
Comparison between the RWM-FN-CP angle of the different groups evaluated. Caption: (A) Comparison by left measurement; (B) Right measurement comparison; (**) p < 0.001. Note: Left measurement analysis was performed by unpaired Student's *t-*test, while right measurement analysis was performed by Mann-Whitney test.

When evaluating the angle formed by the Round Window Membrane on the surface of the Facial Nerve with the Coronal Plane (RWM-FN-CP) by left measurement, we saw that in children the median angle in degrees is reduced compared to the group of adults (p = 0.003) (Fig. 3; Table 3). Likewise, we saw a sharper RWM-FN-CP angle by right measurement in children when compared to adults (p = 0.006) (Fig. 3; Table 3).

**Table 3:** Comparison between the MJR-NF-PC angle of the different groups evaluated.

Group	Average ± SD	Min	Median	Max	p-value
*Right measure*					
Adults	31.5 ± 3.5	24.0	33.0	36.0	0.006^a^
Children	24.7 ± 6.8	16.0	24.0	35.0	
*Left measure*					
Adults	34.9 ± 3.9	29.0	35.0	41.0	0.003^b^
Children	29.1 ± 5.8	21.0	29.5	41.0	

Caption: Values are indicated in degree (°). SD, Standard Deviation; Min, Minimum value found in the group; Max, Maximum value found in the indicated group.

a Statistical value based on the Mann-Whitney test, comparing the group of adults with the group of children.

b Statistical value based on Student's *t*-test, comparing the group of adults with the group of children.

**Fig. 4:**
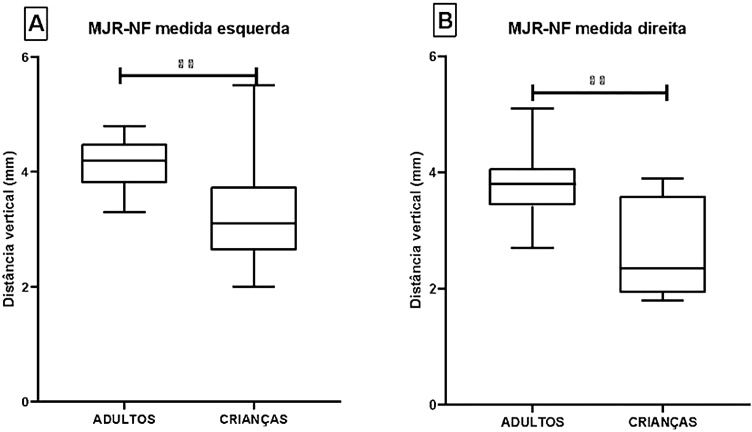
Comparison between the vertical distance between the Round Window Membrane and the Facial Nerve (RWM-FN) of the different groups evaluated. Caption: (A) Comparison by left measurement; (B) Right measurement comparison; (**) p < 0.001. Note: Left measurement analysis was performed by Student's *t*-test, while right measurement analysis was performed by Mann–Whitney test.

When evaluating the vertical distance between the Round Window Membrane and the Facial Nerve (RWM-FN) in millimetres, either by left or right measurement, we saw that in children the median is reduced compared to the group of adults (p = 0.001) (Fig. 4; Table 4).

**Table 4:** Comparison between the MJR-PC vertical distance of the different groups evaluated.

Group	Average ± SD	Min	Median	Max	p-value
*Right measure*					
Adults	3.8 ± 0.5	2.7	3.8	5.1	0.001^a^
Children	2.7 ± 0.9	1.8	2.3	3.9	
*Left measure*					
Adults	4.1 ± 0.5	3.3	4.2	4.8	0.001^b^
Children	3.2 ± 0.9	2.0	3.1	5.5	

Caption: values are indicated in millimeters (mm). SD, Standard Deviation; Min, Minimum value found in the group; Max, Maximum value found in the indicated group.

a Statistical value based on the Mann-Whitney test, comparing the group of adults with the group of children.

b Statistical value based on Student's *t*-test, comparing the group of adults with the group of children.

## Discussion

Severe to profound hearing loss is a prevalent disease worldwide which, when not properly corrected with the use of hearing aids, requires a surgical procedure for insertion of a Cochlear Implant (CI).^8^ Before surgery, obtaining the patient's detailed ear anatomy is essential. Preoperative CT images are essential for the selection of candidates and exclusion of contraindications, and can influence the surgical approach.^10^^,^^11^ Postoperatively, CT can also be used to confirm intracochlear electrode placement, detection of electrode folds, and assessment of electrode integrity.^12^, ^13^, ^14^

Current CI surgery studies aim for a minimal invasive cochlear implantation, reducing the cochlear trauma, and thus possibly leading to best hearing results. For this purpose technologies such as imaging studies and robotic surgeries are being developed.^15^

Access via RW allows full insertion of electrodes with greater precision in the tympanic scale ‒ even in patients with severe anatomical variations in cochlear size and spatial orientation. Therefore, it is believed that this leads to a better outcome in patients' hearing gain. In addition, the insertion of the electrode through the RWM accompanied by good visibility is a relevant factor to reduce intracochlear damage and preserve hearing.^16^^,^^17^ However, the lack of visibility of the RW can make the surgical procedure difficult.^16^

Based on this, the search for ways to predict RW visibility has recently been sought. In this sense, CT helped to establish a relationship between anatomical measurements of the ear and the visualization of the RW, due to its ability to delineate bone anatomy, size of the facial recess and course of the facial nerve within the operative field.^18^ Furthermore, several authors^19^, ^20^, ^21^ have reported differences in the visualization of the RW between adult and pediatric patients. Children have a more restricted Round Window Membrane (RWM) visibility when compared to adults, so that surgical preparation of the RW niche seems to be more demanding in children than in adults.^19^

Thus, we aimed in this study to compare measurements of the RWM and FN between adults and children. This information could help to predict differences in anatomy and possibly in surgery difficulty.

Therefore, in this study, we compared the anatomical measurements of 32 patients (16 adults and 16 children) who underwent CT before CI surgery, in order to verify the RWM-FN-CP angle measurements and vertical distance RWM-FN.

Lloyd et al.^4^ tried to establish a relationship between anatomical measurements of the basal turn of the cochlea and the visualization of the RW during surgery. The aim of these researchers was to investigate changes in cochlear orientation with age and to discuss the implications of any change with respect to CI.

McRackan et al.^5^ demonstrated that it is possible to establish a correlation between the RWM-FN-CP angle, the RWM-FN vertical distance and a greater visibility of the RW at the time of surgery.

Our results show significantly lower angles and distances in children when compared to adults. We observed that the mean RWM-FN-CP angle and RWM-PC vertical distance in children were significantly smaller, for both right and left measurements (angle D: 24.7 ± 6.8 vs. 31.5 ± 3.5; E: 29.1 ± 5.8 vs. 34.9 ± 3.9 and distance D: 2.7 ± 0.9 mm vs. D: 3.8 ± 0.5 mm; E: 3.2 ± 0.9 mm vs. E: 4.1 ± 0.9 mm).

Other radiological parameters can help program CI surgery in children.

Elzayat et al.^22^ assessed the impact of the location of the Chorda Tympani Nerve (CTN) origin on the Round Window (RW) accessibility during pediatric Cochlear Implantation (CI). Authors found that the radiologic CF-SM length (length between the origin of the CTN from the facial nerve to the stylomastoid foramen) of more than 5.4 mm had a powerful prediction capability of the RW inaccessibility.

In another study Elzayat et al.^23^ analysed several radiological features of the facial recess to correlate them with the intraoperative findings to highlight the most reliable predictors of posterior tympanotomy difficulty. The chorda-facial angle, the facial recess aeration, and the chorda-facial to stylomastoid length were respectively the strongest preoperative radiological predictors of the surgical difficulty of posterior tympanotomy during cochlear implantation. A Chorda-facial angle < 25.5 ° was associated with difficult posterior tympanotomy.

The authors^24^ also provided a method to evaluate the Chorda-Facial Angle (CFA) in the HRCT scan. They found a significant-close relation between the CFA and the round window accessibility; the surgical difficulty increased with a need for a modification of the posterior tympanotomy when the angle decreased.

Most recently Elzayat et al.^25^ concluded that preoperative Computed Tomography (CT) predicted the Crista Fenestra (CF) type during cochlear implantation with good sensitivity and accuracy. The CF (crista fenestrae or crista semilunaris) is a sharp bony crest at the anteroinferior boundary of the RW. When the CF is large it has to be partially removed to facilitate electrode insertion.

Barbara et al.^26^ proposed a preoperative radiologic scoring system for predicting Posterior Tympanotomy (PT) and mastoidectomy-associated difficulties during Cochlear Implantation (CI). The radiologic score consisted of 13 radiologic items and was strongly correlated with the surgical difficulty and duration (p < 0.0001). Chorda-facial angle was the strongest predictor, significantly affecting difficulty, surgical duration, and preoperative radiologic score.

Preoperative imaging is mandatory to identify malformations and other anatomical conditions that may be limiting the CI surgical technique and predisposing to complications. Both CT and MRI can identify anomalies in cochlear implant patients, especially in children, but for post lingually deafened adults without conductive or asymmetrical hearing loss, imaging is unlikely to affect surgical decision making.^17^

More studies assessing preoperative imaging and intraoperative findings are paramount for better results in CI surgery.

## Conclusion

We concluded that the RWM-FN-CP angle is more acute, and the RWM-FN vertical distance is smaller in children when compared to adults in preoperative CT of cochlear implant patients.

These measures may help in CI surgery preparation, and inspire future research in this field, developing CT preoperative models and scores.

## ORCID ID

Mayara Risnei Watanabe: 0009-0003-6619-0492

## Funding

None.

## Declaration of competing interest

The authors declare no conflicts of interest.
